# Measuring the outcome and impact of research capacity strengthening initiatives: A review of indicators used or described in the published and grey literature

**DOI:** 10.12688/f1000research.24144.1

**Published:** 2020-06-04

**Authors:** Justin Pulford, Natasha Price, Jessica Amegee Quach, Imelda Bates

**Affiliations:** 1 Department of International Public Health, Liverpool School of Tropical Medicine, Liverpool, L3 5QA, UK

**Keywords:** Research capacity strengthening, Evaluation, Indicators, Review, LMICs

## Abstract

**Background:** Development partners and research councils are increasingly investing in research capacity strengthening initiatives in low- and middle-income countries to support sustainable research systems. However, there are few reported evaluations of research capacity strengthening initiatives and no agreed evaluation metrics.

**Methods:** To advance progress towards a standardised set of outcome and impact indicators, this paper presents a structured review of research capacity strengthening indicators described in the published and grey literature.

**Results:** We identified a total of 668 indicators of which 40% measured output, 59.5% outcome and 0.5% impact. Only 1% of outcome and impact indicators met all four quality criteria applied. A majority (63%) of reported outcome indicators clustered in four focal areas, including: research management and support (97/400), the attainment and application of new research skills and knowledge (62/400), research collaboration (53/400), and knowledge transfer (39/400).

**Conclusions: **Whilst this review identified few examples of quality research capacity strengthening indicators, it has identified priority focal areas in which outcome and impact indicators could be developed as well as a small set of ‘candidate’ indicators that could form the basis of development efforts.

## Introduction

Research capacity strengthening (RCS) has been defined as the “process of individual and institutional development which leads to higher levels of skills and greater ability to perform useful research” ^1^. National capacity to generate robust, innovative and locally appropriate research is considered essential to population health ^2, 3^ and socioeconomic development ^4, 5^. However, wide global disparities in research capacity and productivity currently exist: South Asian countries account for 23% of the World’s population yet produced less than 5% of the global output of scientific publications in 2013 ^6^; and sub-Saharan Africa (accounting for 13% of the global population), contributes 1% of global investment in research and development and holds 0.1% of global patents ^6^. Accordingly, international development partners and research funding bodies are increasingly investing in RCS initiatives in low- and middle-income countries (LMICs). The UK Collaborative on Development Research predicts the United Kingdom’s total aid spend on research will rise to £1.2 billion by 2021 ^7^, a large proportion of which would be direct or indirect investment in RCS in LMICs. The total global spend on RCS in LMICs, while not yet calculated, would likely be many times this figure.

Despite this substantial investment, few robust evaluations of RCS initiatives in LMIC contexts have been presented in the published or grey literatures with the available evidence base characterised by reflective, largely qualitative individual case studies or commentaries ^8^. RCS evaluation frameworks have been described ^9– 11^, but a comprehensive set of standard outcome or impact indicators have not been agreed and common indicators are used inconsistently. For example, publication count has been used as both an output ^12^ and outcome indicator ^13^ sometimes with ^14^ or without ^10^ accounting for publication quality.

The dearth of robust RCS programme evaluation and, more fundamentally, robust evaluation metrics available for consistent application across RCS programmes, has contributed to a paradoxical situation in which investments designed to strengthen the quantity, quality and impact of locally produced research in LMIC settings are themselves hindered by a lack of supporting evidence. As a substantial proportion of RCS investment is derived from publicly funded development assistance ^15– 17^, then ensuring the means to reliably evaluate impact and value for money of research and health system investments assumes even further importance.

This paper aims to advance progress towards the establishment of a standardised set of outcome and impact indicators for use across RCS initiatives in LMIC contexts. As a first step towards this goal, a systematic review of RCS outcome and impact indicators previously described in the published and grey literatures is presented. The review findings highlight the range, type and quality of RCS indicators currently available and allows inconsistencies, duplications, overlaps and gaps to be identified. These results may then be used to inform planning and decision making regarding the selection and/or development of standard RCS evaluation metrics. In the interim, the resulting list of indicators may also serve as a useful resource for RCS programme funders, managers and evaluators as they design their respective monitoring and evaluation frameworks.

## Methods

### Search strategy and study selection

Peer reviewed publications were sought via the following databases: PubMed, Global Health, CINAHL Complete and International Bibliography of the Social Sciences (IBSS). The search was limited to English language publications and was conducted using the keywords: (research capacity) AND (develop* OR build* OR strengthen*) AND (indicator) AND (monitoring OR evaluation). The search was conducted without date limitations up until March 2018. Following removal of duplicates, all retrieved publications were subject to an initial review of the title, abstract and listed keywords. Publications that met, or were suggestive of meeting, the inclusion criteria were then subjected to full text review. Publications subjected to full text review met the inclusion criteria if they: were peer-reviewed; pertained to ‘research capacity’ (as either a primary or secondary focus); and included at least one output, outcome or impact indicator that has been used to measure research capacity or was proposed as a possible measure of research capacity.

The search was supplemented by a manual review of the references listed in each paper that met the final inclusion criteria and by a citation search using first author names for all papers which met the final inclusion criteria from both the initial electronic and supplementary manual searches. A further 19 papers which met the inclusion criteria were identified in this way and included in the review.

**Figure 1.**
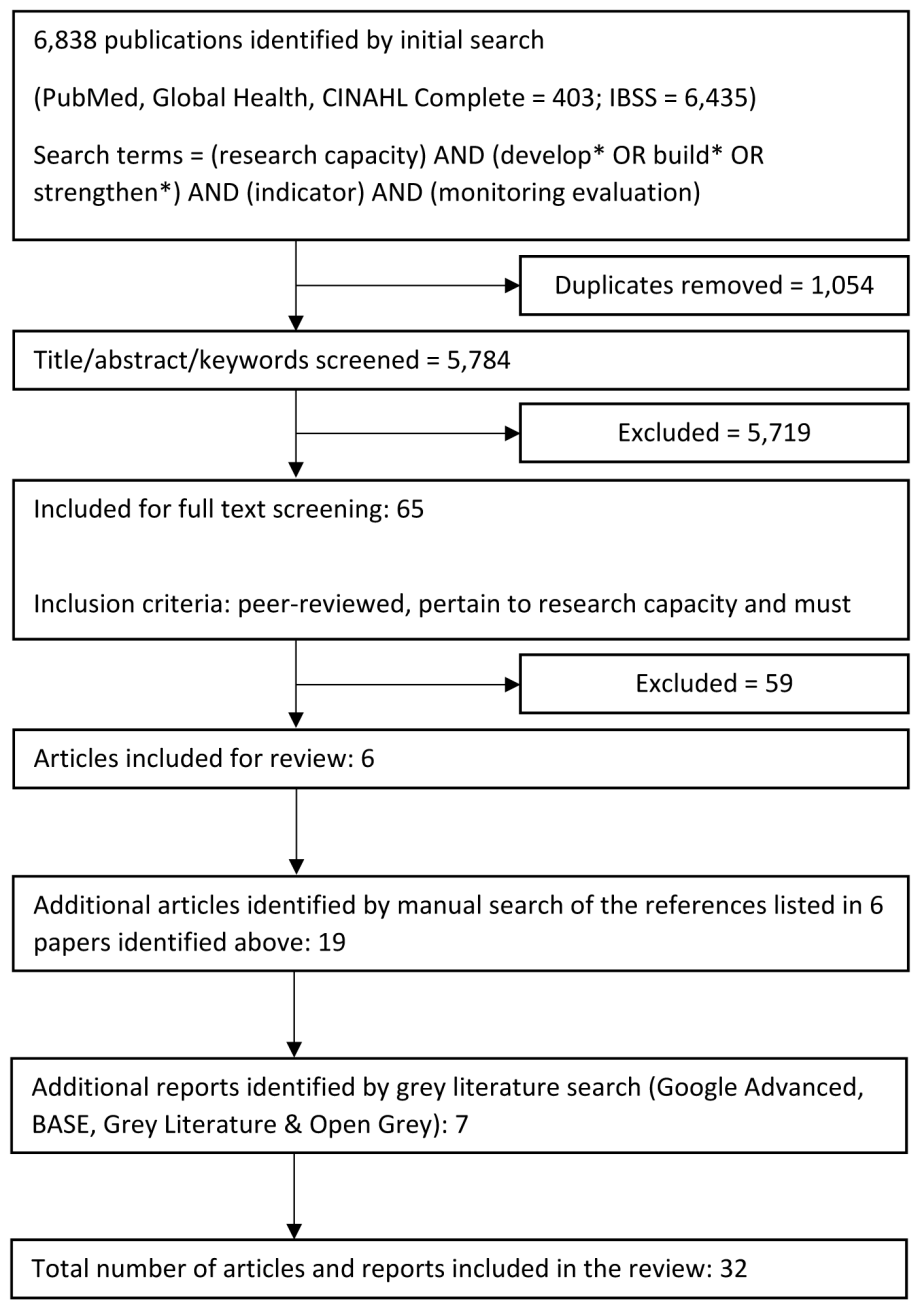
Process and outcomes from literature search strategy.

Relevant grey literature was then sought via the following databases: Google Advanced, BASE, Grey Literature and OpenGrey. The same search terms and inclusion criteria as described above were used. This search was supplemented by a request circulated across the authors’ personal networks for relevant research reports pertaining to RCS evaluation which may fit the inclusion criteria. There were seven reports identified this way, resulting in a final sample of 25 publications and seven reports. Figure 1 depicts the overall process and outcomes from this search strategy.

### Data extraction

Research capacity strengthening indicator descriptions and definitions were extracted from each publication/report and recorded verbatim in an Excel spreadsheet (see *Underlying data*) ^18^. Other information recorded alongside each indicator included: the type of indicator (output, outcome or impact) ( Box 1); the level of measurement (individual research capacity; institutional research capacity; or systemic research capacity); source information (author, year and title of publication/report); and a brief summary of the context in which the indicator was applied. Designation of indicator type (output, outcome or impact) and level of measurement (individual, institutional or systemic) were based on those ascribed by the author/s when reported. Where indicator type and measurement level were not reported, we used our own judgement drawing on the reported context from the respective publication/report.

Some publications/reports used the same indicators across different levels (i.e. as both an individual and an institutional measure) and in these cases we reported the indicator at a single level only based on apparent best fit. However, if the same publication reported the same indicator as both an output and an outcome measure, then it was reported twice. Where there was variation between the way that one publication or another classified an indicator (e.g. the same indicator being described as an ‘output’ indicator in one publication and an ‘outcome’ indicator in another), we remained true to the texts and recorded each separately. Indicators that pertained to the evaluation of course materials or content (e.g. how useful were the PowerPoint slides provided?) were excluded from analysis, although indicators that focused on the outcome of course attendance were retained.

Box. 1 **Defining output, outcome, and impact indicators**

**Output indicators** - defined as measures of programme or project activities that are directly controllable by the RCS initiative (e.g. number of infectious disease experts from country X training in academic writing).

**Outcome indicators** - defined as measures of change in behaviour or performance, in the short- to mid-term, that could reasonably be attributed to the RCS initiative in full or large part (e.g. number of manuscripts published by infectious disease experts from country X following an academic writing course).

**Impact indicators** - defined as measures of longer-term change that may not be directly attributable to the RCS initiative but directly relate to the overarching aims of the RCS initiative (e.g. reduction in infectious disease mortality in country X).

### Data analysis

Once all listed indicators from across the 32 publications and reports had been entered into the Excel spreadsheet, the research team coded all outcome and impact indicators according to their respective focus (i.e. the focus of the indicated measure, such as publication count or grant submissions) and quality. Output indicators were excluded from further analysis. Indicators were coded independently by two researchers, checking consistency and resolving discrepancies through discussion and, if necessary, by bringing in a third reviewer. ‘Focus’ codes were emergent and were based on stated or implied focal area of each indicator. ‘Quality’ was coded against four pre-determined criteria: 1) a measure for the stated indicator was at least implied in the indicator description; 2) the measure was clearly defined; 3) the defined measure was sensitive to change; and 4) the defined measure was time-bound (thus, criteria 2 is only applied if criteria 1 is met and criteria 3 and 4 are only applied if criteria 2 is met).

## Results

### Type and level of identified indicators

**Figure 2.**
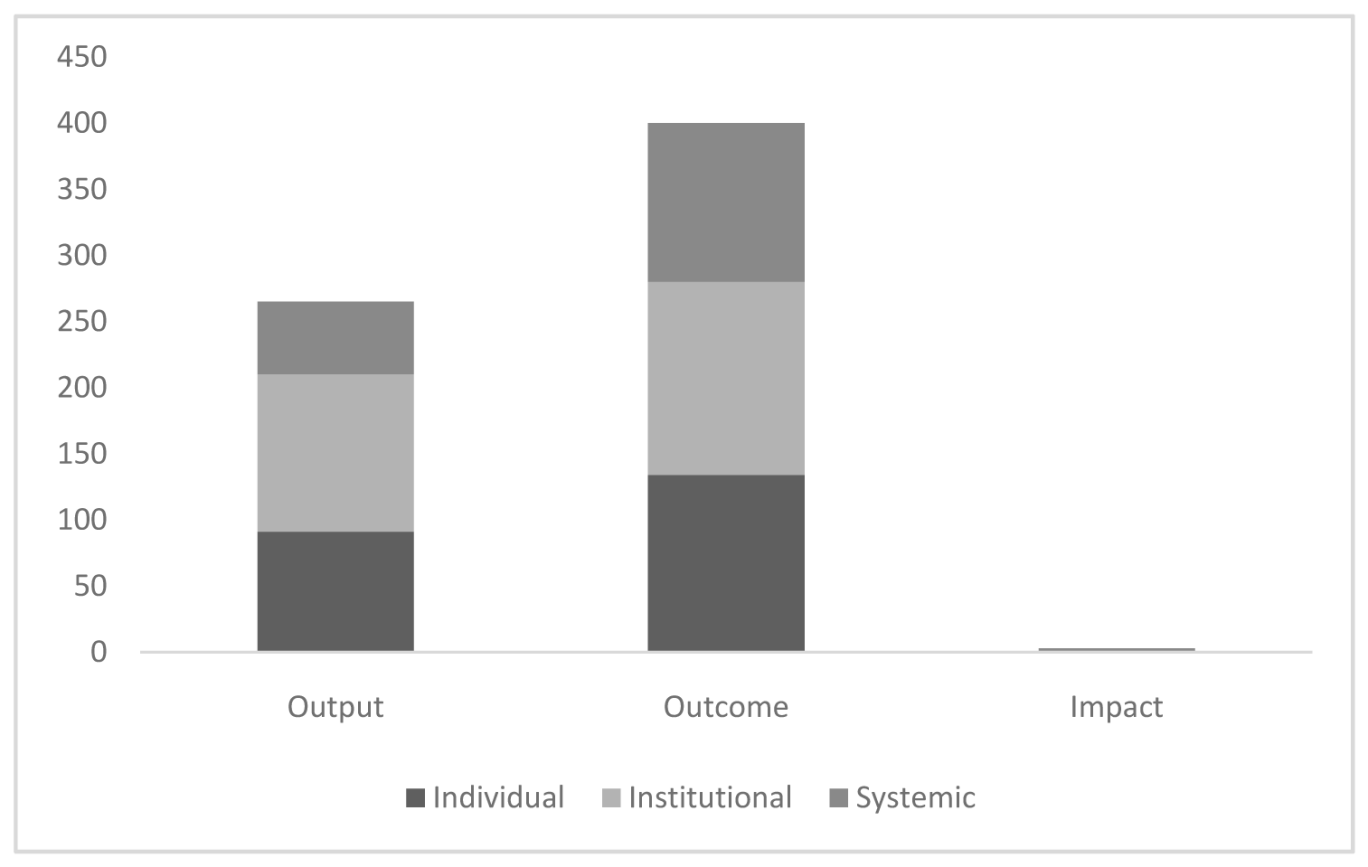
Number of indicators by type and level.

We identified a total of 668 reported or described indicators of research capacity from across the 32 publications or reports included in the review. Of these, 40% (265/668) were output indicators, 59.5% (400/668) were outcome indicators and 0.5% (3/668) were impact indicators. A total of 34% (225/668) of these indicators were measures of individual research capacity, 38% (265/668) were measures of institutional research capacity and 21% (178/668) were systemic measures of research capacity. Figure 2 illustrates the spread of indicator type across these three categories by level. The full list of 668 indicators, inclusive of source information, is available as *Underlying data* ^18^.

### Outcome indicators

**Figure 3.**
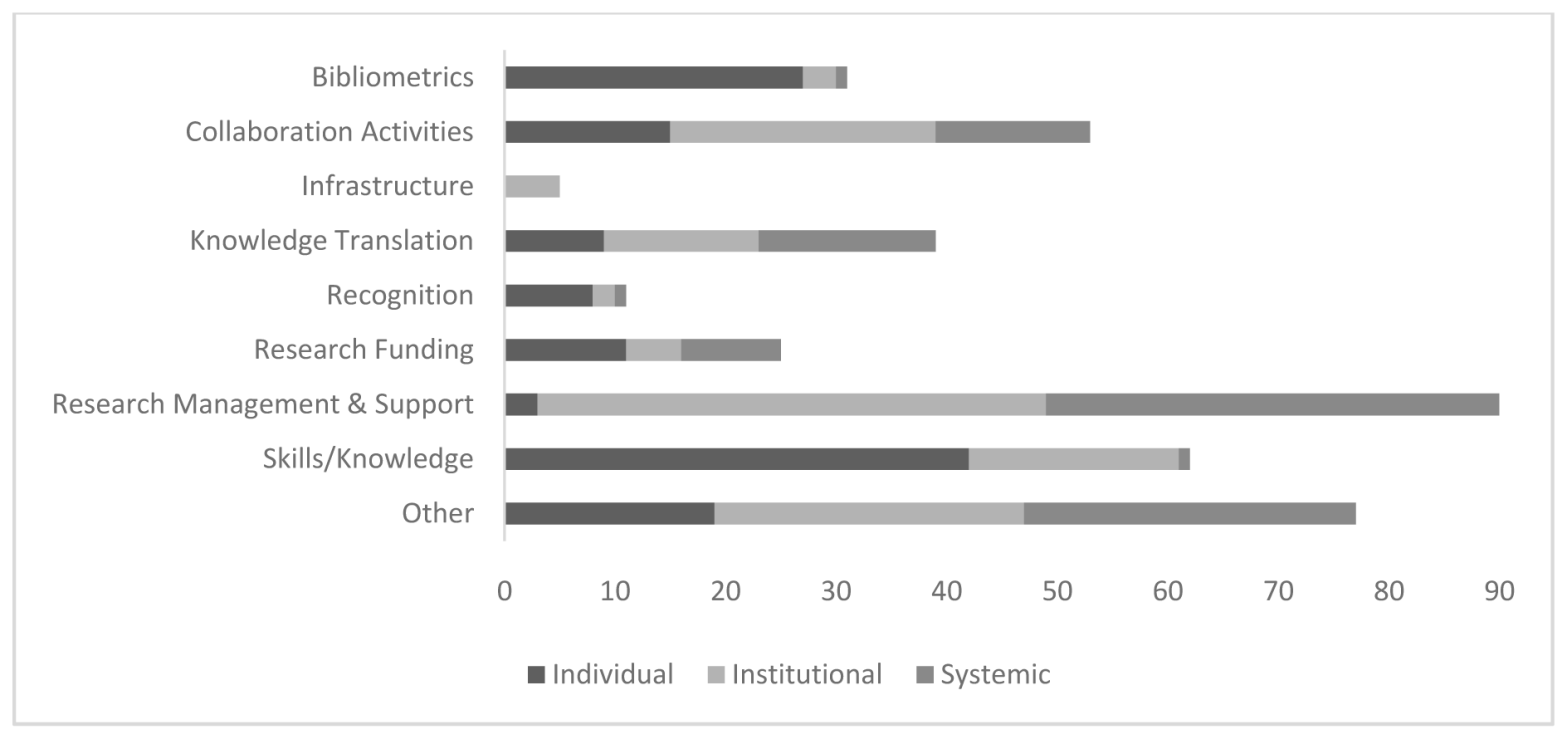
Number of outcome indicators by category and level.

The 400 outcome indicators were subsequently coded to nine thematic categories and 36 sub-categories, as described in Box 2. The categories and the total number of indicators in each (across all three levels) were as follows: research management and support (n=97), skills/knowledge (n=62), collaboration activities (n=53), knowledge translation (n=39), bibliometrics (n=31), research funding (n=25), recognition (n=11), infrastructure (n=5) and other (n=77). Figure 3 depicts the number of outcome indicators by category and level.

Box. 2 **Outcome indicator categories and sub-categories**

**1. Bibliometrics**: Indicators relating to the development, publication and use of written outputs such as peer reviewed journal articles.

    Sub-categories: peer reviewed publication; publication (any form of publication other than peer review); reference (e.g. records of citations); quality (e.g. rating by impact factor).

**2. Collaboration Activities**: Indicators relating to networking, collaborating, mentoring type activities.

    Sub-categories: engagement (evidence of working collaboratively); establishment (creating new networks, collaborations); experience (e.g. perception of equity in a specific partnership).

**3. Infrastructure**: Indicators relating to research infrastructure including buildings, labs, equipment, libraries and other physical resources.

    Sub-categories: suitability (the provision of adequate facilities for research); procurement (e.g. purchase of laboratory equipment).

**4. Knowledge translation**: Indicators relating to the dissemination of research and knowledge, including conferences, media and public education/outreach.

    Sub-categories: dissemination (examples of research being communicated to different audiences); influence (using research knowledge to influence policy, the commissioning of new research, etc).

**5. Recognition**:Indicators relating to professional or institutional esteem.

    Sub-categories: Appointment (e.g. appointed to leadership positions); Awards (i.e. receiving an award); reputation (e.g. invited keynote address).

**6. Research funding**: Indicators relating to funding for research.

    Sub-categories: funds received (e.g. competitive grants); allocation (e.g. allocate budget to support local research); expenditure (use of research funds); access (access to research funding/competitive awards).

**7. Research Management & Support (RMS)**: Indicators relating to the administration of university or research institution systems that make research possible (e.g. finance, ITC and project management).

    Sub-categories: career support (e.g. working conditions, salary and career development); organisation capacity (to manage/support research); research investment; resource access (e.g. to IT, libraries etc); sustainability (of RMS); governance (e.g. formation of ethics review committees); national capacity (to support research); national planning (e.g. developing national research priorities).

**8. Skills/training activities**: Indicators relating to training and educational activities relating to research or research subject area knowledge.

    Sub-categories: attainment (of new skills); application (of new skills); transfer (of new skills).

**9. Other**: Indicators relating to any area other than the eight described above.

    Sub-categories: research quality (e.g. quality of work undertaken); research production (e.g. increase in research activity); research process (e.g. inclusion of new methods or techniques); research workforce (e.g. growth in number of researchers); career advancement (e.g. promotion); equity (e.g. gender equity); miscellaneous.

Table 1– Table 3 present the number of outcome indicators in each sub-category as well as an example indicator for each, by the three respective research capacity levels (individual, institutional and systemic). The category and sub-category designation assigned to all 400 outcome indicators are available as *Underlying data* ^18^.

**Table 1.** Number of individual level outcome indicators by category and sub-variant.

Category	Variant	Number	Example indicator
Bibliometrics	Peer-reviewed publication Publication Reference Quality	5 13 3 6	Number of articles published in peer-reviewed journals ^13^ Number of conference papers ^10^ Citations ^14^ Publications with impact factor indexed in WoS ^21^
Collaboration Activities	Engagement Establishment Experience	10 4 1	Evidence of contribution/membership to networks ^22^ Development of sustainable research collaborations ^23^ Attitudes/behavior are conducive to working effectively in partnership towards development goals ^24^
Knowledge Translation	Dissemination Influence	4 5	Applied dissemination of findings ^22^ Evidence of influence on local strategy & planning ^22^
Recognition	Appointment Awards Reputation	2 3 3	Editor of international/national conference proceedings ^14^ Number of awards/type of awards ^10^ Invitations to speak at meetings ^23^
Research Funding	Funds received	11	New research funding obtained ^23^
RMS	Career support	3	Percent of time spent on research activities ^23^
Skills/knowledge	Application Attainment Transfer	13 27 2	Applying/using new evaluation methodology ^25^ Evidence of progressive skill development ^22^ Shared lessons learned from the distance education program with other personnel at the site ^26^
Other	Research quality Research production Research process Research workforce Career advancement	2 3 8 2 4	Scientific merit of research proposal ^13^ Number of grants completed ^27^ Incorporation of end-users’ concerns into research planning & design ^19^ Evidence that awardees returned to active & independent research in LMICs ^23^ Returned fellows take up leadership roles in scientific networks & communities of practice ^21^

**Table 2.** Number of institutional level outcome indicators by category and sub-variant.

Categories	Variants	Number	Example indicator
Bibliometrics	Peer-review publication Publication Quality	1 1 1	Number of joint scientific publications ^10^ Number of research reports published ^10^ Production of high quality/scientifically sound literature reviews ^25^
Collaboration Activities	Engagement Establishment Experience	15 6 3	Number of joint activities with other research organizations ^10^ Develop research networks within and between institutions ^28^ Collaborations characterized by trust & commitment and continue after award concludes ^23^
Infrastructure	Suitability Procurement	4 1	Facilities and infrastructure are appropriate to research needs and researchers’ capacities ^24^ Research equipment obtained at home institution ^27^
Knowledge Translation	Dissemination Influence	9 5	Number of knowledge exchange events ^10^ Examples of applying locally developed knowledge in strategy policy and practice ^22^
Recognition	Reputation	2	Enhanced reputation & increased appeal of institutions ^19^
Research Funding	Funds Received Allocation Expenditure	3 1 1	Obtaining more funding for research & research skill building training at host organisation ^29^ Budget allocation for specific priority health research areas ^19^ Proportion of funds spent according to workplans ^30^
RMS	Organisational capacity Research investment Career support Resource access Sustainability Governance	14 3 10 4 3 12	Applying data systems for reporting at organizational level ^25^ Funding to support practitioners & teams to disseminate findings ^22^ Evidence of matching novice & experienced researchers ^22^ Access to information technology ^19^ Level of financial sustainability ^10^ Growth & development of institution in line with vision & mission ^31^
Skills/knowledge	Application Attainment Transfer	6 11 2	Applying new skills in financial management to research projects ^25^ Strengthening capacities to carry out methodologically sound evaluations in the South ^25^ Counselling master's and PhD students about appropriate research design and protocols ^32^
Other	Research quality Career advancement Research production Research workforce Research process	5 1 12 6 4	Quality of research outputs ^19^ Evidence of secondment opportunities offered & taken up ^22^ Range & scale of research projects ^19^ Levels of skills within workforce & skill mix of the skills across groups ^22^ Evidence of supporting service user links in research ^22^

**Table 3.** Number of systemic level outcome indicators by category and sub-variant.

Categories	Variants	Number	Example indicator
Bibliometrics	Publication	1	Proportion of TDR grantees' publications with first author [country] institutions ^30^
Collaboration Activities	Engagement Establishment Experience	4 9 1	Changing how organizations work together to share/exchange information, research results ^25^ Partnerships for research dialogue at local, regional & international levels ^23^ TDR partnerships are perceived as useful & productive ^30^
Knowledge Translation	Dissemination Influence	2 14	Media interest in health research ^19^ Policy decision are influenced by research outputs ^24^
Recognition	Reputation	1	Greater Sth-Sth respect between organisations leading to Sth-Sth learning activities ^24^
Research Funding	Allocation Access	6 3	Level of funding of research by the Government ^10^ Local responsive funding access & use ^22^
RMS	National capacity National planning Governance Career support	18 11 18 1	Local ownership of research & health research system evaluation ^19^ Harmonised regional research activities ^23^ Governance of health research ethics ^35^ Researcher salary on par or above other countries in region (by gender) ^10^
Skills/knowledge	Transfer	1	Secondary benefits to students through training, travel & education made them ‘diffusers’ of new techniques between institutions ^23^
Other	Research production Research workforce Research process Equity Research quality Miscellaneous	11 5 7 4 1 2	Generating new knowledge on a research problem at a regional level ^25^ Evidence of brain drain or not ^19^ Several institutions using/applying common methodology to conduct research towards common goal ^25^ Equitable access to knowledge & experience across partnerships ^24^ Proportion of positive satisfaction response from TDR staff ^30^ Importance of multidisciplinary research over the past 5 years ^36^

Table 4 presents the percentage of outcome indicators that met each of the four quality measures as well as the percentage that met all four quality indicators by indicator category. As shown, all outcome indicators implied a measurement focus (e.g. received a national grant or time spent on research activities), 21% presented a defined measure (e.g. had at least one publication), 13% presented a defined measure sensitive to change (e.g. number of publications presented in peer reviewed journals) and 5% presented a defined measure, sensitive to change and time bound (e.g. number of competitive grants won per year). Only 1% (6/400) of outcome indicators met all four quality criteria including: 1) Completed research projects written up and submitted to peer reviewed journals within 4 weeks of the course end; 2) Number of competitive grants won per year (independently or as a part of a team); 3) Number and evidence of projects transitioned to and sustained by institutions, organizations or agencies for at least two years; 4) Proportion of females among grantees/contract recipients (over total number and total funding); 5) Proportion of [Tropical Disease Research] grants/contracts awarded to [Disease Endemic Country] (over total number and total funding); and 6) Proportion of [Tropical Disease Research] grants/contracts awarded to low-income countries (over total number and total funding). Indicators pertaining to research funding and bibliometrics scored highest on the quality measures whereas indicators pertaining to research management and support and collaboration activities scored the lowest.

**Table 4.** Outcome indicator quality by category.

Level	No	Quality measure				All 4 quality measures evident
		Implied	Defined	Sensitive to change	Time-Bound	
		%	%	%	%	%
Bibliometrics	31	100	42	29	6	3
Collaboration Activities	53	100	13	9	0	0
Infrastructure	5	100	20	0	0	0
Knowledge Translation	39	100	18	18	0	0
Recognition	11	100	27	18	0	0
Research Funding	25	100	56	40	12	12
RMS	97	100	7	7	1	1
Skills/Knowledge	62	100	27	0	21	0
Other	77	100	19	19	1	1
**Total**	**400**	**100**	**21**	**13**	**5**	**1**

### Impact indicators

The three impact indicators were all systemic-level indicators and were all coded to a ‘health and wellbeing’ theme; two to a sub-category of ‘people’, one to a sub-category of ‘disease’. The three impact indicators were: 1) Contribution to health of populations served; 2) Impact of project on patients' quality of life, including social capital and health gain; and 3) Estimated impact on disease control and prevention. All three met the ‘implied measure’ quality criteria. No indicators met any of the remaining three quality criteria.

## Discussion

This paper sought to inform the development of standardised RCS evaluation metrics through a systematic review of RCS indicators previously described in the published and grey literatures. The review found a spread between individual- (34%), institutional- (38%) and systemic-level (21%) indicators, implying both a need and interest in RCS metrics across all levels of the research system. This is consistent with contemporary RCS frameworks ^10, 19^, although the high proportion of institutional-level indicators is somewhat surprising given the continued predominance of individual-level RCS initiatives and activities such as scholarship provision, individual skills training and research-centred RCS consortia ^20^.

Outcome indicators were the most common indicator type identified by the review, accounting for 59.5% (400/669) of the total. However, the large number of outcome indicators were subsequently assigned to a relatively small number of post-coded thematic categories (n=9), suggestive of considerable overlap and duplication among the existing indicator stock. Just under two-thirds of the outcome indicators pertained to four thematic domains (research management and support, skills/knowledge attainment or application, collaboration activities and knowledge translation) suggesting an even narrower focus in practice. It is not possible to determine on the basis of this review whether the relatively narrow focus of the reported indicators is reflective of greater interest in these areas or practical issues pertaining to outcome measurement (e.g. these domains may be inherently easier to measure); however, if standardised indicators in these key focal areas are identified and agreed, then they are likely to hold wide appeal.

The near absence of impact indicators is a finding of significant note, highlighting a lack of long-term evaluation of RCS interventions ^8^ as well as the inherent complexity in attempting to evaluate a multifaceted, long-term, continuous process subject to a diverse range of influences and assumptions. Theoretical models for evaluating complex interventions have been developed ^33^, as have broad guidelines for applied evaluation of complex interventions ^34^; thus, the notion of evaluating ‘impact’ of RCS investment is not beyond the reach of contemporary evaluation science and evaluation frameworks tailored for RCS interventions have been proposed ^11^. Attempting to measure RCS impact by classic, linear evaluation methodologies via precise, quantifiable metrics may not be the best path forward. However, the general dearth of any form of RCS impact indicator (as revealed in this review) or robust evaluative investigation ^8, 20^ suggests an urgent need for investment in RCS evaluation frameworks and methodologies irrespective of typology.

The quality of retrieved indicators, as assessed by four specified criteria (measure for the stated indicator was implied by indicator description; measure clearly defined; defined measure was sensitive to change; and defined measure was time-bound) was uniformly poor. Only 1% (6/400) of outcome indicators and none of the impact indicators met all four criteria. Quality ratings were highest amongst indicators focused on measuring research funding or bibliometrics and lowest amongst research management and support and collaboration activities. This most likely reflects differences in the relative complexity of attempting to measure capacity gain across these different domain types; however, as ‘research management and support’ and ‘collaboration activity’ indicators were two of the most common outcome indicator types, this finding suggests that the quality of measurement is poorest in the RCS domains of most apparent interest. The quality data further suggest that RCS indicators retrieved by the review were most commonly (by design or otherwise) ‘expressions’ of the types of RCS outcomes that would be worthwhile measuring as opposed to well defined RCS metrics. For example, ‘links between research activities and national priorities’ ^19^ or ‘ease of access to research undertaken locally’ ^22^ are areas in which RCS outcome could be assessed, yet precise metrics to do so remain undescribed.

Despite the quality issues, it is possible to draw potential ‘candidate’ outcome indicators for each focal area, and at each research capacity level, from the amalgamated list (see *Underlying data*) ^18^. These candidate indicators could then be further developed or refined through remote decision-making processes, such as those applied to develop other indicator sets ^37^, or through a dedicated conference or workshop as often used to determine health research priorities ^38^. The same processes could also be used to identify potential impact indicators and/or additional focal areas and associated indicators for either outcome or impact assessment. Dedicated, inclusive and broad consultation of this type would appear to be an essential next step towards the development of a comprehensive set of standardised, widely applicable RCS outcome and impact indicators given the review findings.

### Limitations

RCS is a broad, multi-disciplinary endeavour without a standardised definition, lexicon or discipline-specific journals ^8^. As such, relevant literature may have gone undetected by the search methodology. Similarly, it is quite likely that numerous RCS outcome or impact indicators exist solely in project specific log frames or other forms of project-specific documentation not accessible in the public domain or not readily accessible by conventional literature search methodologies. Furthermore, RCS outcome or impact indicators presented in a language other than English were excluded from review. The review findings, therefore, are unlikely to represent the complete collection of RCS indicators used by programme implementers and/or potentially accessible in the public domain. The quality measurement criteria were limited in scope, not accounting for factors such as relevance or feasibility, and were biased towards quantitative indicators. Qualitative indicators would have scored poorly by default. Nevertheless, the review findings represent the most comprehensive listing of currently available RCS indicators compiled to date (to the best of the authors’ knowledge) and the indicators retrieved are highly likely to be reflective of the range, type and quality of indicators in current use, even if not identified by the search methodology.

## Conclusion

Numerous RCS outcome indicators are present in the public and grey literature, although across a relatively limited range. This suggests significant overlap and duplication in currently reported outcome indicators as well as common interest in key focal areas. Very few impact indicators were identified by this review and the quality of all indicators, both outcome and impact, was uniformly poor. Thus, on the basis of this review, it is possible to identify priority focal areas in which outcome and impact indicators could be developed, namely: research management and support, the attainment and application of new skills and knowledge, research collaboration and knowledge transfer. However, good examples of indicators in each of these areas now need to be developed. Priority next steps would be to identify and refine standardised outcome indicators in the focal areas of common interest, drawing on the best candidate indicators among those currently in use, and proposing potential impact indicators for subsequent testing and application.

## Data availability

### Underlying data

Harvard Dataverse: Measuring the outcome and impact of research capacity strengthening initiatives: A review of indicators used or described in the published and grey literature - Full listing of retrieved RCS indicators. https://doi.org/10.7910/DVN/K6GIGX ^18^.

This project contains the following underlying data:

- List of RCS Impact Indicators
- List of RCS Outcome Indicators
- List of RCS Output Indicators
- List of Source Documents

Data are available under the terms of the Creative Commons Zero "No rights reserved" data waiver (CC0 1.0 Public domain dedication).
