# Supplementary material for: Measuring the outcome and impact of research capacity strengthening initiatives: A review of indicators used or described in the published and grey literature
Source: F1000Res. 2020 Jun 4;9:517. [Version 1] doi: 10.12688/f1000research.24144.1 (PMC7312283; doi:10.12688/f1000research.24144.1)
Supplement: Peer review history

# Peer review: Reviewer response for version 1

**DOI:** 10.5256/f1000research.26632.r64405

**Published:** 2020-06-29

**Authors:** Meriel Flint-O'Kane

**Affiliations:** 1 Faculty of Public Health and Policy, London School of Hygiene and Tropical Medicine, London, UK

Summary: This paper provides a review and analysis of the quality and suitability of M&E metrics for funding that is allocated to strengthen research capacity in LMIC's. Published and grey literature has been reviewed to identify indicators used to measure the outputs, outcomes and impacts of relevant programmes and the findings have been assessed in terms of content and quality. The authors conclude that the outcome indicators identified were of low quality and impact indicators are almost always missing from RCS MEL frameworks and recommend further work to develop appropriate indicators to measure the outcomes and impacts of research capacity strengthening programmes/activities. Through the review of existing outcome indicators the authors have identified four focal areas against which indicators could be developed.

Methods:

- The search strategy and study selection is clearly described and links to source data are available. Data extraction and analysis methods are also clearly described.

Major:

- No major points to address. This work is by a leading team in the field of RCS research and makes a useful contribution to the literature in providing a thorough review of indicators used to monitor and evaluate work funded to strengthen research capacity.

Minor:

- Though the article is not focused on health research, health is specifically referred to in a few places throughout the article e.g. line 4 (and corresponding references) of the introduction, Research Funding example indicators in Table 2, RMS example indicators in Table 3, numbers 5 and 6 of the 6 outcome indicators meeting all four quality criteria refer to TDR, and the impact indicators are all acknowledged as being specific to health and wellbeing. It would be interesting to understand which other research disciplines featured in the literature reviewed, and the spread of results across disciplines in order that analysis and findings could indicate if there is variety between and/or within disciplines in approaches to MEL for RCS and what can be learnt from this.
- No background, references or justification is given for the pre-determined 'quality' criteria.
- The authors note in 'Limitations' that project documents were not available in the public domain and documents not in English were excluded. Further reflection on to what extent Log Frames, ToC's and other MEL docs  for programmes that have RCS as a primary or secondary outcome would be in the public domain could be helpful e.g. is it common for delivery partners of RCS programmes to make their MEL docs publicly available? If not, are these indicators representative of those currently/recently being used by actors in the RCS programme delivery space or do they represent a subset that is more likely to have publicly available data?

Is the work clearly and accurately presented and does it cite the current literature?

Yes

If applicable, is the statistical analysis and its interpretation appropriate?

Not applicable

Are all the source data underlying the results available to ensure full reproducibility?

Yes

Is the study design appropriate and is the work technically sound?

Yes

Are the conclusions drawn adequately supported by the results?

Yes

Are sufficient details of methods and analysis provided to allow replication by others?

Yes

Reviewer Expertise:

Global health, research capacity strengthening, higher education capacity

I confirm that I have read this submission and believe that I have an appropriate level of expertise to confirm that it is of an acceptable scientific standard.

# Peer review: Reviewer response for version 1

**DOI:** 10.5256/f1000research.26632.r64402

**Published:** 2020-06-24

**Authors:** Francesco Obino, Daniel Fussy

**Affiliations:** 1 Global Development Network, New Delhi, Delhi, India

The article is an timely contribution to an urgent question: how do we know if research capacity strengthening is working? The analysis of the problem (a. the lack of a shared reference framework for evaluating research capacity strengthening, which in turn implies that b. the scope for systematic and cumulative learning remains limited) is convincing and valid. The methodology is clearly explained and up to existing standards and expectations for this kind of exercise. The conclusions are straightforward, and the limitations well articulated (the focus on English, and the bias towards quantitative measures being the most important ones.)

 A few overall comments for the authors, keeping in mind the 'agenda' the article is trying to support (i.e. developing good examples of RCS indicators), and its potential uptake:

1. RCS lack definition too, not just indicators. The article does not differentiate between research capacity strengthening done at the national level and at the international level, or in different fields (health sciences vs social sciences, etc.). While this is key to the aim of the paper to 'survey' existing indicators, the lack of solid evaluation of RCS can be also understood as the result not so much of 'underdevelopment' of the field, but of its overdevelopment in the absence of a shared definition of what RCS is. In this sense, putting all RCS (indicators) in the 'same box' might in fact reinforce the confusion around what is there to be measured, and how. International donor-funded, project-based RCS efforts differ (in scope, objectives and means) from the RCS effort of a science council or a local research training institution - despite overlaps. Often, the difference in objectives might make indicators hard to include in the same box. In this sense, the paper should acknowledge the lack of a shared definition of RCS, and the limitation it poses to an analysis of indicators. For this specific article, it might be useful to define RCS as international, donor-funded, project-based set of activities. Arguably, the very need of a discussion on RCS evaluation is largely driven by the fact that RCS is part of the evaluation-heavy international donor sector. This might help further defining the relevant timeframe for the search, and situating RCS historically.
2. RCS is more than the sum of quality outputs. I wonder about the lack of discussion on 'process indicators' given the nature of RCS as a set of activities. These are notoriously difficult (but not impossible) to use in the donor-funded, project-based, time-bound RCS efforts, but might be very relevant to describe change and ultimately impact.
3. RCS impacts research systems, policy, or development? When it comes to discussion of impacts and impact indicators, the lack of definition of RCS becomes an insurmountable limitation. The study could acknowledge the need for unpacking the link between output, outcome and impact measurement/definition (particularly in light of lack of shared definition of RCS) in internationally funded programs, as a complementary exercise to the surveying of indicators. The fact that the very few impact indicators identified reveal an expectation for RCS to deliver impact on population health outcomes is a good example of the limitations imposed by lack of clear definitions.
4. How important is the UK? Given the global audience of the piece, it might be useful to explain why the figures relating to projected RCS funding from the UK are significant to describe larger trends - particularly if figures include both 'direct' and 'indirect' RCS.

Is the work clearly and accurately presented and does it cite the current literature?

Yes

If applicable, is the statistical analysis and its interpretation appropriate?

Yes

Are all the source data underlying the results available to ensure full reproducibility?

Yes

Is the study design appropriate and is the work technically sound?

Yes

Are the conclusions drawn adequately supported by the results?

Yes

Are sufficient details of methods and analysis provided to allow replication by others?

Yes

Reviewer Expertise:

Research capacity building methodologies, political theory, international relations

We confirm that we have read this submission and believe that we have an appropriate level of expertise to confirm that it is of an acceptable scientific standard.

# Peer review: Reviewer response for version 1

**DOI:** 10.5256/f1000research.26632.r64403

**Published:** 2020-06-23

**Authors:** Peter Taylor

**Affiliations:** 1 Institute of Development Studies, Brighton, UK

The article addresses an issue that is receiving renewed interest in recent years - research capacity strengthening (RCS), and the particular challenge of evaluating outputs, outcomes and impacts of RCS initiatives.

 The study undertook a structured review of RCS indicators in the published and grey literature. Key findings included the identification of rather few examples of quality RCS, with emphasis on four focal areas (research management and support, skill and knowledge development, research collaboration and knowledge transfer. The study concludes that there is significant room for development of indicators, and consequently the potential adoption of these to allow a more systematic approach to RCS approaches and to their subsequent evaluation.

 The study is clearly presented and has a solid methodology. The validity of the findings rest on the extent to which the systematic review did identify published material that engages in this issue. As the authors note, it is likely that there is a wider body of grey literature in the form of project and program reports that were not located through the search. This suggests that there is need for more published work on this topic (making the paper therefore relevant and useful), and perhaps reinforces a wider view that many RCS efforts are inadequately evaluated (or not evaluated at all). An earlier World Bank Institute report on evaluation of training (Taschereau, 2010 ^1^), for example, had highlighted challenges in evaluation of the impact of training and institutional development programs. The study refers briefly to RCS interventions, taking training as an example, but this only related to training which makes up a small percentage of the overall efforts towards RCS.

 It would be very interesting to situate this welcome study in the context of broader discussions and debates on RCS, particularly as a contribution to theory and practice at strengthening research capacity at individual, organizational and system levels. The latter of these is the most complex to conceptualise, to implement, and to measure, and is receiving valuable attention from RCS stakeholders such as the Global Development Network (GDN, 2017 ^2^) through their Doing Research Program - a growing source of literature for subsequent review.

 As the authors of the study note, there is a danger in identifying RCS indicators that are seen as having universal application and attractiveness because they are relatively easy to measure. There is an equal, related danger that, due to relative measurability, a majority of RCS interventions become so streamlined in terms of their approach that they begin to follow recipe or blueprint approaches.

 The study is agnostic on different approaches to RCS. Work undertaken by the Think Tank Initiative (TTI) for example (Weyrauch, 2014 ^3^) has demonstrated a range of useful RCS approaches, including flexible financial support, accompanied learning supported by trusted advisors/program officers, action learning, training and others. In a final evaluation of the Think Tank Initiative (Christoplos *et al.*, 2019 ^4^), training was viewed as having had the least value amongst several intervention types in terms of RCS outcomes, whilst flexible financial support and accompanied learning processes were viewed as being significantly more effective. It would be interesting to identify indicators of outcomes or even impacts that might relate to different types of RCS interventions which were not included in the publications reviewed by this study.

 A key indicator of RCS identified by the TTI evaluation, which interestingly does not appear explicitly in the indicator list of this study, was leadership. As the authors indicate, there are likely to be other valuable indicators not surfaced through this review and this requires more work.

 This study offers a very important contribution to a field currently being reinvigorated and is highly welcome. Rather than being valued because it may potentially offer a future blueprint list of indicators, (not least since, as the authors observe, the indicator list generated in this study is partial in comparison to a much wider potential range), its value lies particularly in its potential for contribution to further debate and dialogue on the theory and practice of RCS interventions and their evaluation; this dialogue can in turn be further informed by access to a more diverse set of grey literature and by engagement with stakeholders who have experience and interest in strengthening this work. Hopefully the authors of this study, and other researchers, will continue this important line of work and promote ongoing discussion and debate.

Is the work clearly and accurately presented and does it cite the current literature?

Yes

If applicable, is the statistical analysis and its interpretation appropriate?

Yes

Are all the source data underlying the results available to ensure full reproducibility?

Yes

Is the study design appropriate and is the work technically sound?

Yes

Are the conclusions drawn adequately supported by the results?

Yes

Are sufficient details of methods and analysis provided to allow replication by others?

Yes

Reviewer Expertise:

International development, organizational learning and development, research capacity strengthening

I confirm that I have read this submission and believe that I have an appropriate level of expertise to confirm that it is of an acceptable scientific standard.

## References

[1] : Evaluating the impact of training and institutional development programs - a collaborative approach (English). *EDI Learning Resources Series, World Bank Institute (WBI)*.2010; Reference source

[2] : Doing Research Assessments: Understanding Research Systems in Developing Countries. *Global Development Network: New Delhi*.2017; Reference source

[3] : Capacity Development within the Think Tank Initiative. *Think Tank Initiative: Ottawa*.2014; Reference source

[4] : External Evaluation of the Think Tank Initiative (TTI) Phase Two, 2014-2019. *NIRAS: Allerød*.2019; Reference source

# Peer review: Reviewer response for version 1

**DOI:** 10.5256/f1000research.26632.r64400

**Published:** 2020-06-23

**Authors:** Erica Di Ruggiero

**Affiliations:** 1 Dalla Lana School of Public Health, University of Toronto, Toronto, ON, Canada

- The article outlines clear research questions and methods and provides a very useful summary of findings from a structured review of research capacity strengthening indicators for use in low- and middle-income countries (LMICs). Terminology is overall quite clearly defined. Some greater precision relate to the use of the terminology: context for indicator's application; knowledge transfer vs. knowledge translation are used.
- The definition of knowledge translation provided seems limiting and perhaps the authors meant only transfer. It would have been helpful to have some descriptive data on the sources of the publications/reports from which the indicators were derived (i.e. were they all published by academics vs. any from research funders). For example, it's unclear if any indicators developed by funders such as the International Development Research Centre and others that support LMIC research are included.
- The limitations section is clear.
- It would have been helpful to have the authors elaborate a bit more on the dearth of qualitative indicators, appreciating the fact that they would have 'scored poorly by default' because of the methodology used. Could the authors comment in the conclusion on areas for indicator development (like qualitative indicators; equity-related indicators - for e.g. I note that perception of equity in a specific partnership was part of the definition for collaboration and in the 'other' category, but to my knowledge, equity didn't really appear elsewhere)?

Is the work clearly and accurately presented and does it cite the current literature?

Yes

If applicable, is the statistical analysis and its interpretation appropriate?

Yes

Are all the source data underlying the results available to ensure full reproducibility?

Yes

Is the study design appropriate and is the work technically sound?

Yes

Are the conclusions drawn adequately supported by the results?

Yes

Are sufficient details of methods and analysis provided to allow replication by others?

Partly

Reviewer Expertise:

Public health research; evaluation

I confirm that I have read this submission and believe that I have an appropriate level of expertise to confirm that it is of an acceptable scientific standard.
